# Adolescents’ health and well-being into the COVID-19 pandemic: A two-wave prospective investigation– The HUNT study

**DOI:** 10.1007/s00127-025-02978-1

**Published:** 2025-08-18

**Authors:** Kirsti Kvaløy, Erik Reidar Sund, Tormod Rimehaug, Kristine Pape, Jo Magne Ingul, Vegar Rangul

**Affiliations:** 1 HUNT Research Centre, Department of Public Health and Nursing, Faculty of Medicine and Health Sciences, Norwegian University of Science and Technology, Levanger, Norway; 2 Centre for Sami Health Research, Department of Community Medicine, Faculty of Health Sciences, UiT The Arctic University of Norway, Tromsø, Norway; 3 Levanger Hospital, Nord-Trøndelag Hospital Trust, Levanger, Norway; 4 Regional Centre for Child and Youth Mental Health and Child Welfare, Department of Mental Health, Faculty of Medicine and Health Sciences, Norwegian University of Science and Technology, Trondheim, Norway; 5 Department of Public Health and Nursing, Faculty of Medicine and Health Sciences, Norwegian University of Science and Technology, Trondheim, Norway

**Keywords:** Young-HUNT, Adolescents, Loneliness, Mental distress, Health, Life quality, COVID-19

## Abstract

**Purpose:**

Using data on Norwegian adolescents, this study aimed to explore changes in mental health, quality of life, somatic health complaints and loneliness from before and one year into the COVID-19 pandemic, also considering the changes according to socioeconomic position (SEP).

**Methods:**

The study involved a cross-sectional comparative design with data from Young-HUNT4 (2017–2019) (*n* = 4347) and Young-HUNT COVID (May/June 2021) (*n* = 2033), aged 16–19 years. Additionally, longitudinal changes from Young-HUNT4 (*n* = 1565), aged 13–15 years, with follow-up in Young-HUNT COVID were explored. The impact of SEP was investigated through regression analyses and investigating prevalence changes in high and low SEP groups.

**Results:**

In the cross-sectional comparison, boys and girls reported higher levels of loneliness and mental distress (boys only) into the pandemic compared to before, while general health and quality of life remained stable. Longitudinally, all factors changed adversely except for general health in boys. Comparing younger (13–15 years) with older (16–19 years) adolescents from Young-HUNT4, demonstrated the same adverse pattern as in the longitudinal sample. Poor health, poor quality of life and loneliness were more prevalent in the low compared to the high SEP group. In the low SEP group, mental distress, poor general health and life quality worsened in boys while improved in girls during the study period.

**Conclusion:**

Except for mental distress in boys, general health and life quality did not deteriorate in the study period, although loneliness increased in both sexes. In the low SEP group, girls seemed to cope better than boys where health and well-being even improved.

**Supplementary Information:**

The online version contains supplementary material available at 10.1007/s00127-025-02978-1.

## Introduction

*Most countries in the world implemented full or partial school closures in the period from 2020 to 2021 related to the coronavirus disease 2019 (COVID-19) pandemic. To prevent further spread of the SARS-CoV-2 virus and adverse impacts on health in the population*,* this resulted in social distancing and lockdown efforts that negatively affected health and well-being of children and adolescents. This growing recognition was* *mostly based on cross-sectional investigations. The Young-HUNT COVID data collection in combination with earlier data collected prior to the COVID-19 pandemic from the same Norwegian adolescent population*,* allowed* *us to study this in a longitudinal perspective gaining potential general knowledge of underlying factors influencing the adverse health trends observed among today’s young people.*

Global estimates said that up to 1.5 billion of the ones younger than 20 years were out of school in early 2020 and again in early 2021 related to the COVID-19 pandemic restrictions [1]. In Norway, all schools were mandatory closed down from March 12, 2020, and then there were dynamic changes throughout 2020 and 2021 that sometimes differed geographically [2]. Compared to many European countries, Norway is sparsely populated and has better social welfare benefit arrangements. These factors combined may have resulted in different conditions for the young people in Norway compared to other equivalent regions of the Western world.

Adolescence is a critical period in life concerning psychosocial development characterized by social transition regarding individual role, peer relationships and family cohesion [3]. This makes adolescents especially vulnerable to the disruptive changes imposed by the pandemic restrictions as mental distress seems to be associated with stress from social isolation [4–6]. Over the last decades there has been several reports of an increase in young people feeling unhappy, reporting mental distress [7–10], and loneliness [11]. How the COVID-19 pandemic interacted with these trends may have varied regionally and globally due to the differences in pandemic restrictions and dissimilarities in society and population [12]. According to the World Health Organization (WHO), the global prevalence of anxiety and depression increased by a massive 25% in the first year of the COVID-19 pandemic [13]. Most studies investigating the impact of the pandemic on mental health have described an increase in mental distress [1, 14–17], but not all [4–6].

Norway implemented a nationwide lockdown on March 12, 2020. This involved compulsory closure of schools and cancellation of organized sport activities. Within a few weeks after the first closure, which also encouraged restrictive social interactions, dynamic change in infection and insecurity regarding the consequences of the contagion, resulted in various restrictions throughout 2020 and 2021. Norwegian adolescents suffered from more restrictions than younger children with repeated and extended periods of home schooling and cancelled sports activities throughout the pandemic period [18]. Norwegian adolescents also experienced adverse mental health during the pandemic [18–20]. In the study by Hafstad et al., somatic health complaints were shown to increase ahead of and 15 months into the pandemic whereof girls appeared to be more affected than boys [18]. Other studies also report a more adverse effect on mental health in girls compared to boys [17, 21, 22]. Most studies published concerning the COVID-19 pandemic’s effect on adolescents have been based on cross-sectional data not being able to shed light on the longitudinal effects within the same group of individuals. Even if such studies are of immense benefit, the effect of ageing within adolescence is of uttermost importance to consider as shown in a previous Norwegian study [23].

The COVID-19 pandemic led to societal changes concerning the use of technologies; digital means of learning and communication, and an increase in screen time usage [1], which may have influenced the experience of loneliness [24]. Others have also suggested loneliness to be an important trigger of the increase in mental health problems, quality of life and somatic complaints during the pandemic [18, 24, 25]. An increase in loneliness among adolescents was observed even before the pandemic [11]. This could suggest that the trend might be a continuation of pre-existing patterns rather than solely a result of the pandemic. Furthermore, the age-typical developmental changes must also be controlled for to clarify the influence of the pandemic. However, it is important to consider that the pandemic might have exacerbated these trends due to increased social isolation and stress. The interplay of multiple contributing factors requires complex datasets that enable the necessary complex designs and analytic strategies to fully understand the dynamics at play.

Social inequality impact mental health acting as additional strain or protective factors affecting the influence of the COVID-19 pandemic on mental health and quality of life. Those with low socioeconomic status (families with low education levels or limited financial resources) may have been affected more [17], hence, important to address with special emphasis in a longitudinal setting like ours.

Based on current knowledge concerning the consequences of the COVID-19 pandemic on the adolescent population our hypothesis was that adolescent health and well-being deteriorated as an effect of the public measures that was imposed and that this was enhanced by low SEP. To study the potential consequences of the pandemic on Norwegian youth, we compared a cohort where data were collected before the pandemic outbreak (2017-19) and after the major close-down of the Norwegian society, combined with comparable cohort data collected in the same population a few decades earlier.

## Methods

### Study populations

The population-based Trøndelag Health Study (HUNT) was initiated in 1984–1986 (HUNT1) and consists of three subsequent waves (HUNT2-4) conducted every 11th year (24, 25). In the HUNT Study, inhabitants (aged 13 +) of Trøndelag County in the central part of Norway have been invited to participate. The data collection from adolescents, the Young-HUNT Study (26, 27), was first carried out in 1995–1997 with the Young-HUNT1 Survey (YH1, *n* = 8980). It consisted of data from 13–19-year-olds where the youngest were invited to a follow-up, Young-HUNT2 (YH2, *n* = 2427), in 2000–2001. Cross-sectional data from new adolescent cohorts were collected in 2006–2008, Young-HUNT3 (YH3, *n* = 8199), and in 2017-19, Young-HUNT4 (YH4, *n* = 8066). The surveys were conducted at secondary schools lower and upper. Pupils completed a questionnaire during one school hour. Specially trained personnel visited all the schools for interviews, clinical measurements and collection of biological samples (YH3 and YH4).

The HUNT COVID Study was started in 2021 with the main purpose of studying risk factors, severity, and the consequences of the nationwide control measures on public health. Data from adolescents, the Young-HUNT COVID Survey (YHC), was conducted in May/June 2021. All pupils from the upper secondary schools in the whole Trøndelag County (*n* = 32) aged 16 + were invited. An electronic questionnaire was sent out mainly to be answered in one school hour. Many questions were the same as in YH4, but we also included questions specifically concerning the COVID-19 pandemic. Due to teachers’ strikes, cancelled exams and the closing–down of certain schools, participation rates, 16–19-year-olds, only reached 35% (*n* = 5034). In the same geographical region as YH4, participation rate was 40% (*n* = 1778).

The study samples included in the present study consisted of both cross-sectional data of YH4 and YHC, and longitudinal data from YH4 to YHC and YH1 to YH2. The cross-sectional data from 16–19-year-olds in YH4 (*n* = 4347, 51% girls) and YHC (*n* = 2033, 59% girls) were used to compare trends. In addition, the youngest participants (13–15-year-olds) from YH4 (*n* = 3719, 51% girls) were compared to the oldest (16–19-year-olds) YH4 (*n* = 4347, 51% girls) to investigate the effects of age on the health measures used.

**Fig. 1:**
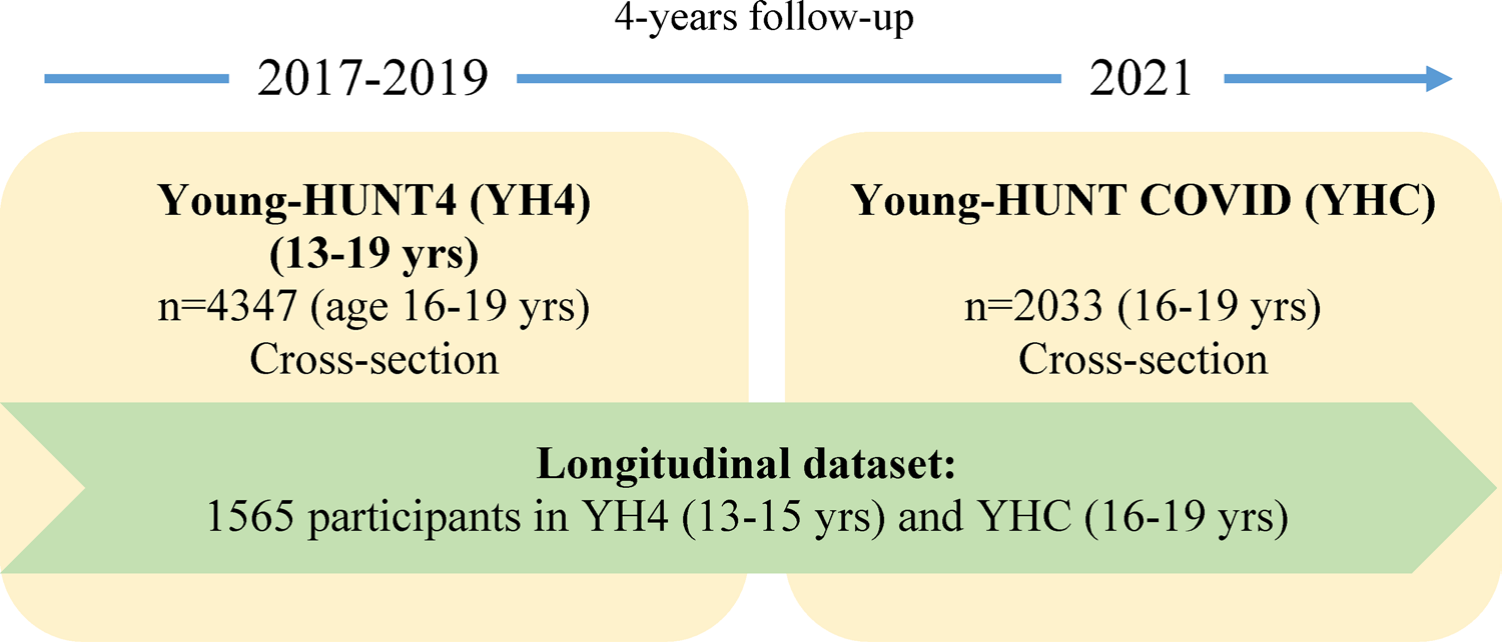
Flow chart of the study populations, Young-HUNT4, YH4 (2017–2019) and Young-HUNT COVID, YHC (2021); cross-sectional datasets (age 16–19 years) and a longitudinal dataset consisting of 1565 individuals participating at age 13–15 years and in a follow-up four years later at age 16–19 years

Longitudinal analyses to study changes from YH4 to YHC comprised 1565 participants (61% girls). The longitudinal YH1 to YH2 dataset, used as comparison as the follow-up years in the two are comparable, consisting of 2399 participants (54% girls). For study designs, see Fig. 1 and S1.

To assess the potential impacts of the low participation rate in the follow-up in YHC, participants in both waves were compared descriptively at the age of 13–15 years at baseline participation (YH4) (data not shown).

### Study variables

*Socioeconomic position (SEP)* was deduced from the variable self-perceived family affluence measured using the single question *‘*How well off do you think your family is compared to most others” with three response options that were dichotomized into 1) High (“About the same as most others*”*,* “*Better financial situation) and 2) Low (Worse financial situation). *Loneliness* was measured by the single question ‘Are you lonely?’ rated on a 5-point scale was dichotomized into 1) not lonely (Very rarely/Rarely/Sometimes lonely) and 2) lonely (Often/Very often). *General health* was measured by the question *‘*How is your health at the moment*?’* rated on a 4-point scale ranging from “Poor” to 4 “Very good” and further dichotomized into 1) Good/Very good and 2) Poor/Very poor. The 10-item Hopkins Symptom Checklist (HSCL-10), a shorter version of the longer SCL-25 scales [26], assessed symptoms of anxiety and depression (mental distress). The HSCL-10 is a widely used tool for measuring psychological distress and symptoms of depression. Studies have shown that HSCL-10 has good internal consistency [27], which makes it a reliable tool for assessing psychological distress over time.

In our dataset, the internal consistency was satisfactorily with a Cronbach’s alpha of 0.92 in both YH4 and YHC. Each item was rated on a four-point Likert scale ranging from 1 (‘Not bothered’) to 4 (‘Very much bothered’). As with previous evaluations of the score [28], participants who had responded to more than eight of the ten questions were also included [29], (*n* = 154 YH4, and *n* = 37 in YHC). An average item score of ≥ 1.85 was used to identify symptoms of mental distress, following suggestions from Strand et al. [28].

The instrument ILC - *Inventory of Life Quality* [30] was used for measuring quality of life. The ILC-28-score is a reliable way of measuring life quality [30]. The translated Norwegian version used here is validated [31]. The score contains seven questions with answers on a 5-point Likert scale from “Very good” to “Very poorly”. The coded responses are summarized in a sumscore from 0 to 28, where 0 is high and 28 is low quality of life [30, 32]. In our dataset, the internal consistency was satisfactorily with a Cronbach’s alpha of 0.85 in both YH4 and YHC. ILC was also dichotomized with sex-specific upper 25% percentile cut-offs, ≥ 11 in girls and ≥ 8 in boys.

### Statistics

Differences between the two study samples (YH4 and YHC) were tested by Pearson’s Chi-square tests for categorical variables and T-tests for continuous variables. The Friedman test was used for the longitudinal analyses. The results are shown in either means with standard errors or as proportions, number of participants and percentages. Two-tailed p-values < 0.05 were considered statistically significant. To explore the association between self-perceived family affluence (proxy for SEP) and various health outcomes and whether these differed pre- and into the pandemic, regression analyses were performed as binary logistic for the categorical outcomes (loneliness and general health) and linear regression for continuous outcomes (mental distress and life quality). These were performed adjusted for sex and age (Model 1) and in addition for survey participation (YH4 and YHC) (Model 2). Estimated effects are presented as Odds ratios (ORs) and standardized betas (Bs), both with 95% confidence intervals and p-values. Difference between YH4 and YHC concerning SEP was additionally tested by calculating prevalence ratios and differences. To analyse drop-off statistics, YH4 participants that were invited but did not take part in the YHC follow-up (830 boys and 703 girls) where compared to the ones that did participate. Statistically, boys did not differ, but the non-participant girls reported slightly lower life quality (measured with ILC) at YH4 than their peers. To confirm our findings in the longitudinal analyses, we additionally used STATA specifying random effects panel models to examine changes in outcomes across survey waves and between sexes, including interaction terms for wave and sex. Analyses were restricted to participants who participated in both YH4 and YHC, and standard errors were clustered at the individual level. For self-perceived family affluence, loneliness and self-reported general health, we applied logistic regression (xtlogit) and report predicted probabilities (as percentages) using the margins postestimation command. For HSCL-10 and ILC, we used linear regression (xtreg) and estimated marginal means using the margins command. To assess sex-specific changes from YH4 to YHC, we used the lincom command. These analyses were run in two models where the second was adjusted for time-varying age.

## Results

**Fig. 2:**
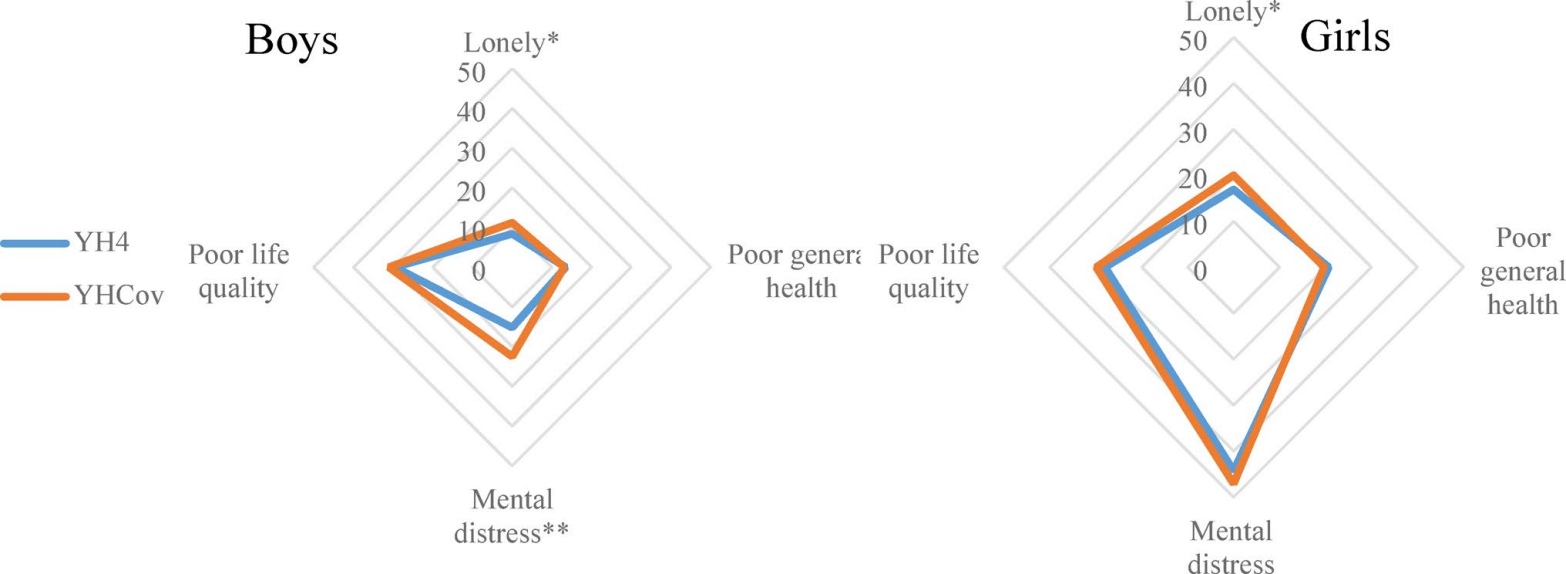
Proportions (%) of participants reporting loneliness, poor general health, poor life quality and mental distress in the two cross-sections: Young-HUNT4, YH4 (2017–2019) and Young-HUNT COVID, YHC (2021) in boys and girls. * P value < 0.05, ** P value < 0.001 indicating statistically significant difference between YH4 and YHC

Girls participated in higher rates compared to boys in YHC (59%) in contrast to YH4 (51%), and the adolescents were slightly older, 17.7/17.8 and 17.5 years, YHC and YH4 respectively (Table 1). In both surveys, the prevalence of loneliness, poor general health, mental distress and life quality were higher in girls than in boys. Loneliness was more prevalent in YHC compared to YH4 in both boys and girls (8.4% vs. 11.2% in boys and 16.9% vs. 20% in girls), which represent a negative change of 2.8% in boys and 3.1% in girls. Interestingly, only boys and not girls reported poorer metal health from pre- to-pandemic conditions (Table 1; Fig. 2). This represented an increase in the HSCL-10 sumscore of 1.12 units. Perception of SEP (self-perceived family affluence) seemed to be unchanged comparing pre-pandemic with pandemic data (Table 1).

**Table 1:** Characterisation and sex-stratified comparison of the Young-HUNT4, YH4 (2017–2019) and Young-HUNT COVID, YHC (2021) cross-sections

Variables	YH4	YHC	*P* value *	YH4	YHC	*P* value *
	Boys (*n* = 2135)	Boys (*n* = 828)		Girls (*n* = 2212)	Girls (*n* = 1205)	
Age: Mean, SD	17.5 (1.0)	17.7 (0.9)	< 0.001	17.5 (0.9)	17.8 (0.9)	< 0.001
Family affluence: Low, n (%)	176 (8.4)	80 (9.7)	0.252	244 (11.1)	140 (11.7)	0.648
Loneliness: Often/Very often, n (%)	171 (8.4)	90 (11.2)	0.021	362 (16.9)	237 (20)	0.025
General health: Poor/Very poor, n (%)	286 (13.5)	108 (13.1)	0.758	455 (20.7)	237 (19.7)	0.493
HSCL-10 sumscore, SD	14.24 (5.0)	15.36 (6.0)	< 0.001	19.46 (7.4)	19.6 (7.1)	0.536
ILC sumscore, SD	5.65 (4.2)	5.84 (4.4)	0.369	7.99 (4.6)	8.32 (4.8)	0.073

*T-test for continuous and Pearson Chi square for categorical variables. Missing in YH4: Family affluence (self-perceived) (*n* = 60), Loneliness (*n* = 165), General health (*n* = 31), HSCL (*n* = 284), ILC (*n* = 403). Missing in YHC: Family affluence (self-perceived) (*n* = 10), Loneliness (*n* = 43), General health (*n* = 4), HSCL (*n* = 83), ILC (*n* = 563).

**Fig. 3:**
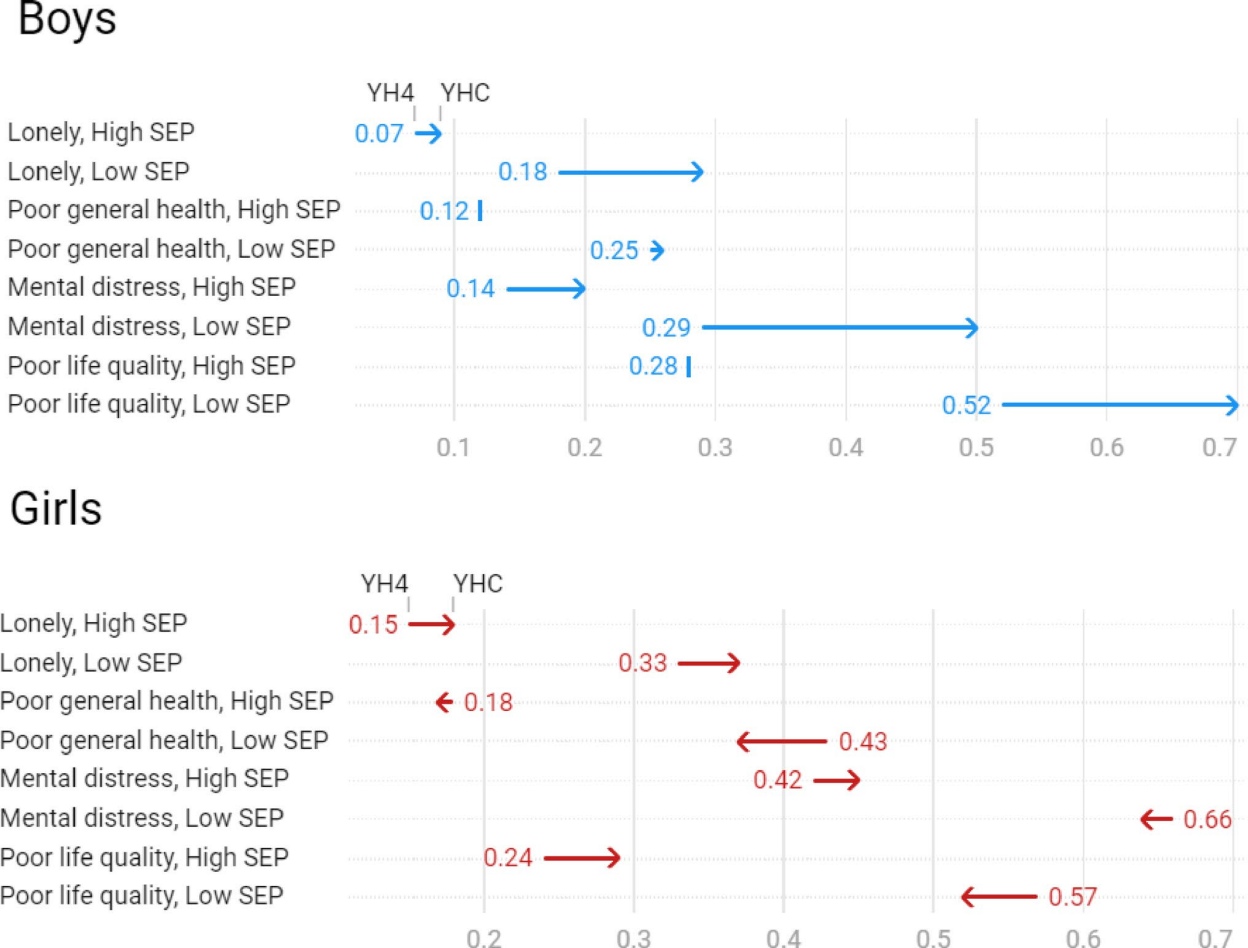
Sex-specific prevalence of loneliness, poor general health, mental distress and poor life quality in the Young-HUNT4, YH4 (2017–2019) and Young-HUNT COVID, YHC (2021) cross-sections stratified on socioeconomic position (SEP - family affluence). Lonely (Often/Very often), Poor mental health (Poor/Very poor), Mental distress (HSCL-10 ≥ 1.85), Poor life quality (ILC, sex-specific 25% upper percentile cut-offs; ≥ 8 in boys and ≥ 11 in girls)

In a combined YH4/YHC dataset, regression analyses demonstrated that low SEP negatively affected all the selected outcome variables (Table S1). To explore whether the impact of SEP on health and well-being changed during the pandemic, sex-stratified prevalence ratios were calculated (Table S2). In the high SEP group, the prevalence of poor general health and life quality stayed unchanged in boys and nearly unchanged in girls, while the other two outcomes deteriorated. In the low SEP group, all the outcomes worsened in boys, while loneliness was the only one that developed adversely in girls (Fig. 3).

Following the younger adolescents (13–15-year-olds) longitudinally (from 2017 to 2019 to May/June 2021) displayed a different pattern than the one observed comparing the cross-sections (YH4 and YHC). In both sexes, self-reported low family affluence (4.7–8.1% in boys and 6.9–10.8% in girls), loneliness (5.5–9.0% in boys and 12.1–20.4% in girls) and mental distress (HSCL-10 caseness) (14.3–24.3% in boys and 34.5–52.0% in girls) increased. Poorer health (11.6–19.5%) and lower life quality (ILC) (mean (SD) 7.3 (4.6) to 8.36 (4.8)) were only observed in girls (Table 2).

**Table 2:** Characterisation and sex-stratified comparison of the Young-HUNT4, YH4 (2017–2019) and Young-HUNT COVID, YHC (2021) longitudinal dataset (*n* = 1565)

Variables	Boys (*n* = 605)			Girls (*n* = 960)		
	YH4	YHC	*P* value *	YH4	YHC	*P* value *
Age: Mean, SD	14.6 (1.0)	17.8 (0.9)		14.6 (1.0)	17.9 (0.9)	
Family affluence: Low, n (%)	28 (4.7)	49 (8.1)	0.006	66 (6.9)	103 (10.8)	< 0.001
Loneliness: Often/Very often, n (%)	32 (5.5)	53 (9.0)	0.024	113 (12.1)	192 (20.4)	< 0.001
General health: Poor/Very poor, n (%)	64 (10.7)	69 (11.4)	0.513	111 (11.6)	187 (19.5)	< 0.001
HSCL-10 sumcore, SD	14.00 (6.9)	15.43 (5.9)	< 0.001	17.66 (6.9)	20.48 (7.4)	< 0.001
ILC sumscore, SD	5.47 (4.2)	5.84 (4.4)	0.126	7.30 (4.6)	8.36 (4.8)	< 0.001

*Friedman test. Missing: Family affluence (self-perceived) (*n* = 20), Loneliness (*n* = 47), General health (*n* = 13), HSCL-10 (*n* = 77), ILC (*n* = 116)

To confirm and examine these results, additionally analyses examining changes across survey waves (YH4 and YHC) and between sexes, including interaction terms for wave and sex specifying random effects panel models, were performed. The results (Model 1) agreed very well with our previous findings, except for boys reporting poorer life quality (ILC) over time (see Table S3).

The observed discrepancy between the YH4 compared to the YHC cross-sections with the YH4-follow-up longitudinal sample could be due to age, as older adolescents tend to report more adverse health than younger adolescents do. To investigate this, differences in the younger and older age groups (13–15 vs. 16–19-year-olds) of the YH4 (total sample, *n* = 8066) was explored (Table S4). As presumed, we could confirm that in both sexes older compared to younger adolescents reported more loneliness, poorer general health, and higher mental distress. These findings were confirmed in Model 2 using random effects models adjusting for the variation in age due to time between YH4 and YHC (see Table S3).

In addition, the same age-related trend of adverse self-reported health was seen when comparing longitudinal data from an earlier Young-HUNT follow-up survey (*n* = 2399); baseline in 1995-97 (YH1) with follow-up four years later in 2000-01(YH2) (see FigS1 and Table S5). For the earlier Young-HUNT survey dataset (YH1 with follow-up in YH2), adolescents in the age range 16–19 years in YH1 (not participating in the YH2 follow-up) was compared to YH2 participants to evaluate differential trends over time in that five-year period. In general, no adverse developments were observed concerning loneliness, general and mental health, although girls were slightly lonelier in 1995–1997 (YH1), than in 2000–2001 (YH2) (see Fig S2).

## Discussion

Most studies have shown that health protection efforts during the COVID-19 pandemic such as repeated quarantine, confinement and lockdowns have negatively affected especially mental health in children and adolescents [33]. Even so, countries and geographical regions were subject to variations in the capacity to handle the situation and strictness in measurements, which would affect the populations differently. Norway represents a wealthy society with well-organized social benefit arrangements; hence restrictions were most probably less intrusive compared to some of the other populations which other studies were based on. In addition, our study population inhabit a rural area in Norway not as densely populated as the ones represented in many other COVID-19 focused studies.

In our study, data from the same region in Norway were collected pre-pandemic (2017–2019) and again well into the pandemic (May/June 2021) which gave us the unique possibility of studying factors related to the pandemic in the same adolescent population over time. The main findings of our study based on primarily two cross-sections of 16–19-year-olds living in the same area previously and under the COVID-19 pandemic were generally that the adolescents were not affected as negatively as could be expected; general health, quality of life and the adolescents’ perception of family affluence seemed to have stayed unchanged. The rise in loneliness observed also before the pandemic, however, seemed to have continued into the pandemic. Interestingly, only boys and not girls reported poorer mental health from the time before the pandemic compared to into the pandemic, which is the opposite of what others have shown and deviate from the negative trends identified before the pandemic where girls compared to boys reported the most adverse development [9, 10, 34].

In contrast to the findings in the two cross-section, adverse general and mental health, quality of life and loneliness were identified longitudinally at the two time points pre-pandemic (2017–2019) and four years later into the pandemic at follow-up (May/June 2021) in a dataset consisting of 1565 individuals. Independent of the pandemic, the same negative development was observed in an earlier Young-HUNT based longitudinal dataset from 1995 to 1997 with follow-up in 2000–2001. Our suggestion is that the adverse health developments seen in the longitudinal sample spanning the period pre-pandemic into the pandemic is not a result of the pandemic restrictions, but rather a consequence of the increase in age from early until later adolescence as have been evident from other longitudinal studies [23, 35].

There has been a constant rise in loneliness observed globally over the last 20 years among young people with special emphasis on females [11]. The same trend has been identified in Norway [36, 37]. Young individuals are especially vulnerable to loneliness as they are in a state of social, psychosocial and cognitive transition from childhood to adulthood [38]. The increased loneliness prevalence causes public health concerns, as it is associated with risk factors such as suicidal behaviour [39, 40], social anxiety and depression [41] as well as future adult psychological illness and poor health outcomes [42]. It is therefore of immense importance to investigate whether the restrictions during the pandemic gave further rise in loneliness, with the associated adverse consequences for health and well-being. There is a suggestion that this negative trend is linked to the parallel rise in digital media usage such as social media and smart phones [11] which seems to have been further strengthened during the COVID-19 pandemic [43–45].

In our study, loneliness was more prevalent during the pandemic compared to prior to the pandemic in both sexes. This is in line with another Norwegian study, which show that loneliness had increased linearly from 2014 into the pandemic in 2021 [20]. Contrary to this, Hansen et al., identified no additional adverse change in loneliness during the pandemic in young Norwegian adults (18–24 years [46]. This was suggested to be because adolescents’ relationships with parents and peers during the pandemic did not seem to deteriorate [20].

In a recent review of studies concerning mental health in Europe during the COVID-19 pandemic, a general increase in mental health problems related to the pandemic was observed [47–49], although the findings are mixed [33]. By late 2020, i.e. after the most intrusive lock-down period, the increase appeared to have slowed down probably due to less fear and uncertainty amongst people. In our study, one of the most interesting findings was that only boys and not girls reported poorer mental health from the time before the pandemic compared to June 2021. This is in disagreement with the findings from a Finish study [50] and other Norwegian studies [18, 20] where mental distress seemed to worsen more in girls compared to boys. These discrepancies despite common Nordic conditions presumably being quite homogenous may be due to lack of total comparability concerning degree of urbanity. As mentioned, the population of the northern part of Trøndelag representing the sample in our study lack large cities, whilst the other Norwegian sample referred to [20] is nationally representative; hence with more participants inhabiting urban and more densely populated areas. A study that nicely describes the impact of different lockdown trajectories is the one by Meyer and colleagues where data are collected from two Australian states, Victoria, and Queensland [51].

We found based on the cross-sectional comparison of the 16–19-year-olds pre- and under the COVID-19 pandemic that general health, quality of life and the adolescents’ perception of family affluence seemed to have stayed unchanged. This could be because the restrictions in general did not have such negative effects on health as would be expected, or that the most stringent restrictions had been lifted at the point of data collection (May/June 2021) and conditions had normalised. As shown in a Dutch study, levels of loneliness, general mental health and life satisfaction declined initially in the pandemic but then improved to previous levels when restrictions were more relaxed [52]. This has also been shown by others who have suggested that the decline is a result of increased resilience in the population who adapted to the situation [53] and also were influenced by the positive effects of collective solidarity [54].

The fact that general health and life satisfaction stayed unchanged probably also depended on the adolescents perceiving their household financial situation to be the same as before. Financial decline related to the pandemic has in previous studies been shown to have negative impacts on physical and mental health in adolescents [55, 56], whereof several demonstrate that girls’ mental health was more adversely affected than boys’ in more vulnerable socioeconomic situations during the pandemic [57, 58].

In general, girls seem to be more prone to develop mental health problems than boys and factors influencing this are suggested to involve imposed expectations, social roles [59] and stronger stress reactivity [60]. Even if the proportion of girls compared to boys were shown to report adverse health and well-being generally, girls in our study did not develop negatively in the COVID-19 pandemic period. In fact, girls in the low SEP group seemed to be better off than before the pandemic. Family support and school contentment is important for mental health and well-being [61] and school pressure and coping in daily life are causes of poor life satisfaction and mental health [62, 63], also pinpointed by many adolescent girls themselves in Norway. As the effect of school pressure most probably changed in the pandemic period, it is tempting to speculate on the potential positive influence on girls this may have had. In a British survey, one-third of the students (aged 8–18) reported improvement in their mental wellbeing during the first COVID-19 lockdown and these were more likely than their peers to report improvement concerning factors such as school, relational, and lifestyle [6]. The authors suggest the reduction in school stress or learning systems during the pandemic to be better suited for some. Another issue interesting to emphasise related to our findings is the worrying trend of school refusals and “flight” from public schools among children and adolescents in Norway which has increased after the pandemic [64] and the mechanisms related to this [65]. Even if the pandemic resulted in disadvantages such as enhanced outsideness and reduced social skills [64], it is tempting to speculated whether life outside the school atmosphere also were beneficial for some adolescents.

In conclusion, our study indicate that the pandemic did not lead to a decline in the overall health and quality of life in adolescents, except for the increase in loneliness in both sexes and mental distress among boys. In general, girls appeared to cope better than boys, with health and life quality even showing improvement among girls from families with lower socioeconomic position. Understanding the contextual factors that contributed to this positive development and finding ways to sustain this situation beyond the pandemic is crucial for preventing further deterioration of mental health and well-being among adolescents in the future.

## Strengths and limitations

The major strength of this study is the ability to compare the situation concerning adolescents’ health and well-being prior to and into the pandemic in the same population. The HUNT Study is a large population-based study largely representative of Norwegians [66, 67], although it does not include large city inhabitants. The population is relatively homogenous, with a low migration rate. Nearly everyone attends the same public schools and benefits from the same welfare and public health services although it may vary marginally between municipalities. The observed negative mental health and loneliness trends [9, 37] agree with both national and global circumstances [11, 20, 68].

There are several limitations to the study, one being that data is based on self-report. Another drawback is the low participation rate in the Young-HUNT COVID Survey. As the variations in the school’s participation rates were due to the randomness of teachers that were on strike, regional lockdowns, and flexible times at school because of cancelled exams, reduced representativeness is not likely an important issue. In addition, confirming main coincident characteristics in the baseline study (YH4) age-groups 13–15 years, comparing with non-participants in the follow-up (Young-HUNT COVID), precluded the presence of selection bias and strengthened our findings. In our study, the cut-off level defining statistically significant findings was set to p-value < 0.05. However, if we were to compensate for potential type I errors due to multiple hypotheses tested, a cut-off value could have been set to 0.01 (Bonferroni Corrections accounting for five main hypotheses 0.05/5 = 0.01, α_new_ = α_original_/n). This would result in the observed increase in loneliness from Young-HUNT4 to Young-HUNT COVID not being present anymore. However, the increased prevalence concerning loneliness is still of interest as our study is primarily a descriptive one.

Anyhow, the influence due to variation in lockdown measures would be difficult to precisely account for as different regions and municipalities implemented different types of lockdown measures. At some points nationwide lockdown were declared, whereas some regions advocated for social distancing and limitations on gatherings rather than legally enforcing a lockdown.

In this study, SEP was measured by a question concerning self-perceived family affluence. Most studies using subjective SEP demonstrate a positive impact of higher SEP on better health outcomes in youth [69]. Interestingly, this association has also been shown to be reciprocal, i.e. that mental distress could negatively affect the subjective perception of SEP [70], but this direction of association depends on the health outcome. Concerning self-rated health for instance, the observed direction of association seems to be that SEP affects later health and not vice versa [71]. An alternative measure of SEP could have been the objective measure of parental education, which would in our case require family linkage through Statistics Norway. Using parental or family SEP as a proxy for adolescent SEP in predicting health outcomes may however be debatable since adolescents’ own perception of social status seem to impact health the most [72]. Even so, based on their meta-analysis findings, Quon and McGrath recommend incorporating both subjectively and objectively measured SEP in combination to better understand mechanisms underlying health disparities.

A limitation in our study could be that such a complex experience as loneliness was assessed by a single question both in YH4 and YHC. Although, it has been shown that single-item measures are reliable, correlate highly with multi-item scales and are useful measures of loneliness [73].

## Supplementary Information

Below is the link to the electronic supplementary material.

Supplementary Material 1

## Data Availability

Due to restrictions imposed by the HUNT Research Centre, in accordance with the Norwegian Data Inspectorate’s guidelines, data cannot be made publicly available. The data is currently stored in the HUNT Databank, and there are restrictions for handling data files. Data may be available upon request to the HUNT Data Access Committee (hunt@medicine.ntnu.no). The HUNT data access information (available at http://www.ntnu.edu/hunt/data) describes in detail the policy regarding data availability.

## References

[1] Viner R, Russell S, Saulle R, Croker H, Stansfield C, Packer J et al (2022) School closures during social lockdown and mental health, health behaviors, and well-being among children and adolescents during the first COVID-19 wave: a systematic review. JAMA Pediatr 176(4):400–409. 10.1001/jamapediatrics.2021.5840. (**PubMed PMID: 35040870**) 35040870 10.1001/jamapediatrics.2021.5840

[2] Forsberg JT, Thorvaldsen S (2022) The severe impact of the COVID-19 pandemic on bullying victimization, mental health indicators and quality of life. Sci Rep 12(1):22634 Epub 20221231. 10.1038/s41598-022-27274-9 36587112 10.1038/s41598-022-27274-9 PMC9804241

[3] Viner RM, Ross D, Hardy R, Kuh D, Power C, Johnson A et al (2015) Life course epidemiology: recognising the importance of adolescence. J Epidemiol Community Health 69(8):719–720 Epub 20150202. 10.1136/jech-2014-205300 25646208 10.1136/jech-2014-205300 PMC4515995

[4] Cost KT, Crosbie J, Anagnostou E, Birken CS, Charach A, Monga S et al (2022) Mostly worse, occasionally better: impact of COVID-19 pandemic on the mental health of Canadian children and adolescents. Eur Child Adolesc Psychiatry 31(4):671–684 Epub 20210226. 10.1007/s00787-021-01744-3 33638005 10.1007/s00787-021-01744-3 PMC7909377

[5] van der Velden PG, van Bakel HJA, Das M (2022) Mental health problems among Dutch adolescents of the general population before and 9 months after the COVID-19 outbreak: a longitudinal cohort study. Psychiatry Res 311:114528. 10.1016/j.psychres.2022.114528 35344687 10.1016/j.psychres.2022.114528 PMC8942449

[6] Soneson E, Puntis S, Chapman N, Mansfield KL, Jones PB, Fazel M (2023) Happier during lockdown: a descriptive analysis of self-reported wellbeing in 17,000 UK school students during Covid-19 lockdown. Eur Child Adolesc Psychiatry 32(6):1131–1146 Epub 20220217. 10.1007/s00787-021-01934-z 35174418 10.1007/s00787-021-01934-z PMC8853175

[7] Bommersbach TJ, McKean AJ, Olfson M, Rhee TG (2023) National trends in mental health-related emergency department visits among youth, 2011–2020. JAMA 329(17):1469–1477. 10.1001/jama.2023.4809 10.1001/jama.2023.4809 PMC10155071 37129655

[8] Twenge JM. Increases in Depression, Self-Harm, and, Suicide Among US (2020) Adolescents After 2012 and Links to Technology Use: Possible Mechanisms. Psychiatr Res Clin Pract.;2(1):19–25. Epub 20200909. 10.1176/appi.prcp.20190015. PubMed PMID: 36101887; PubMed Central PMCID: PMCPMC9176070 10.1176/appi.prcp.20190015 PMC9176070 36101887

[9] Krokstad S, Weiss DA, Krokstad MA, Rangul V, Kvaløy K, Ingul JM et al (2022) Divergent decennial trends in mental health according to age reveal poorer mental health for young people: repeated cross-sectional population-based surveys from the HUNT study, Norway. BMJ Open 12(5):e057654 Epub 20220518. 10.1136/bmjopen-2021-057654 35584877 10.1136/bmjopen-2021-057654 PMC9119156

[10] Keyes KM, Platt JM (2024) Annual research review: sex, gender, and internalizing conditions among adolescents in the 21st century - trends, causes, consequences. J Child Psychol Psychiatry 65(4):384–407. 10.1111/jcpp.13864 37458091 10.1111/jcpp.13864 PMC12341061

[11] Twenge JM, Haidt J, Blake AB, McAllister C, Lemon H, Le Roy A (2021) Worldwide increases in adolescent loneliness. J Adolesc 93(1):257–269. 10.1016/j.adolescence.2021.06.006 34294429 10.1016/j.adolescence.2021.06.006

[12] Fountoulakis KN, Karakatsoulis GN, Abraham S, Adorjan K, Ahmed HU, Alarcón RD et al (2022) The effect of different degrees of lockdown and self-identified gender on anxiety, depression and suicidality during the COVID-19 pandemic: data from the international COMET-G study. Psychiatry Res 315:114702 PubMed PMID: 35839639; PubMed Central PMCID: PMCPMC9247180 35839639 10.1016/j.psychres.2022.114702 PMC9247180

[13] WHO (2022) COVID-19 pandemic triggers 25% increase in prevalence of anxiety and depression worldwide. https://www.who.int/news/item/02-03-2022-covid-19-pandemic-triggers-25-increase-in-prevalence-of-anxiety-and-depression-worldwide [updated 2 March] PMC9998057 35414629

[14] Houghton S, Kyron M, Lawrence D, Hunter SC, Hattie J, Carroll A et al (2022) Longitudinal trajectories of mental health and loneliness for Australian adolescents with‐or‐without neurodevelopmental disorders: the impact of COVID‐19 school lockdowns. J Child Psychol Psychiatry 63(11):1332–1343. 10.1111/jcpp.13579 35194802 10.1111/jcpp.13579 PMC9790479

[15] Villanti AC, LePine SE, Peasley-Miklus C, West JC, Roemhildt M, Williams R et al (2022) COVID-related distress, mental health, and substance use in adolescents and young adults. Child Adolesc Ment Health 27(2):138–145. 10.1111/camh.12550 35253363 10.1111/camh.12550 PMC9018497

[16] Pustake M, Mane S, Ganiyani MA, Mukherjee S, Sayed M, Mithbavkar V et al (2022) Have the COVID-19 pandemic and lockdown affected children’s mental health in the long term? A repeated cross-sectional study. BMJ Open 12(7):e058609 Epub 20220707. 10.1136/bmjopen-2021-058609 35798530 10.1136/bmjopen-2021-058609 PMC9263377

[17] Ravens-Sieberer U, Kaman A, Erhart M, Devine J, Schlack R, Otto C (2022) Impact of the COVID-19 pandemic on quality of life and mental health in children and adolescents in Germany. Eur Child Adolesc Psychiatry 31(6):879–889 Epub 20210125. 10.1007/s00787-021-01726-5 33492480 10.1007/s00787-021-01726-5 PMC7829493

[18] Hafstad GS, Sætren SS, Wentzel-Larsen T, Augusti EM (2022) Changes in adolescent mental and somatic health complaints throughout the COVID-19 pandemic: A Three-Wave prospective longitudinal study. J Adolesc Health 71(4):406–413 Epub 20220617. 10.1016/j.jadohealth.2022.05.009 35725540 10.1016/j.jadohealth.2022.05.009 PMC9212438

[19] Lehmann S, Skogen JC, Sandal GM, Haug E, Bjørknes R (2022) Emerging mental health problems during the COVID-19 pandemic among presumably resilient youth -a 9-month follow-up. BMC Psychiatry 22(1):67 Epub 20220127. 10.1186/s12888-021-03650-z 35086520 10.1186/s12888-021-03650-z PMC8793097

[20] von Soest T, Kozák M, Rodríguez-Cano R, Fluit DH, Cortés-García L, Ulset VS et al (2022) Adolescents’ psychosocial well-being one year after the outbreak of the COVID-19 pandemic in Norway. Nat Hum Behav.;6(2):217–28. Epub 20220120. 10.1038/s41562-021-01255-w. PubMed PMID: 35058644 10.1038/s41562-021-01255-w 35058644

[21] Ertanir B, Kassis W, Garrote A (2021) Longitudinal changes in Swiss adolescent’s mental health outcomes from before and during the COVID-19 pandemic. Int J Environ Res Public Health. 10.3390/ijerph182312734 34886458 10.3390/ijerph182312734 PMC8656984

[22] Källmen H, Hallgren M (2024) Mental health problems among adolescents during the COVID-19 pandemic: a repeated cross-sectional study from Sweden. Scand J Public Health 52(3):329–335. 10.1177/14034948231219832 38217316 10.1177/14034948231219832 PMC11067385

[23] Hafstad GS, Sætren SS, Wentzel-Larsen T, Augusti EM (2021) Adolescents’ symptoms of anxiety and depression before and during the Covid-19 outbreak - A prospective population-based study of teenagers in Norway. Lancet Reg Health Eur 5:100093 PubMed PMID: 34557820; PubMed Central PMCID: PMCPMC8454857 34557820 10.1016/j.lanepe.2021.100093 PMC8454857

[24] Guazzini A, Pesce A, Gino F, Duradoni M (2022) How the COVID-19 Pandemic Changed Adolescents’ Use of Technologies, Sense of Community, and Loneliness: A Retrospective Perception Analysis. Behav Sci (Basel).;12(7). Epub 20220713. 10.3390/bs12070228. PubMed PMID: 35877298; PubMed Central PMCID: PMCPMC9311528 10.3390/bs12070228 PMC9311528 35877298

[25] Houghton S, Kyron M, Hunter SC, Lawrence D, Hattie J, Carroll A et al (2022) Adolescents’ longitudinal trajectories of mental health and loneliness: the impact of COVID‐19 school closures. J Adolesc 94(2):191–205. 10.1002/jad.12017 35353417 10.1002/jad.12017 PMC9087620

[26] Derogatis LR, Lipman RS, Rickels K, Uhlenhuth EH, Covi L (1974) The Hopkins symptom checklist (HSCL): a self-report symptom inventory. Behav Sci 19(1):1–15.3830190102. PubMed PMID: 4808738 4808738 10.1002/bs.3830190102

[27] Haavet OR, Sirpal MK, Haugen W, Christensen KS (2011) Diagnosis of depressed young people in primary health care–a validation of HSCL-10. Fam Pract 28(2):233–237. 10.1093/fampra/cmq078 20937663 10.1093/fampra/cmq078

[28] Strand BH, Dalgard OS, Tambs K, Rognerud M (2003) Measuring the mental health status of the Norwegian population: a comparison of the instruments SCL-25, SCL-10, SCL-5 and MHI-5 (SF-36). Nord J Psychiatry 57(2):113–118 PubMed PMID: 12745773 12745773 10.1080/08039480310000932

[29] Søgaard ABI, Tell G, Røysamb E (2003) A comparison of the CONOR mental health index to the HSCL-10 and HADS. Measuring mental health status in the Oslo health study and the Nord-Trøndelag health study. Norsk Epidemiologi 13(2):279–284

[30] Jozefiak T, Larsson B, Wichstrøm L, Wallander J, Mattejat F (2010) Quality of life as reported by children and parents: a comparison between students and child psychiatric outpatients. Health Qual Life Outcomes 8:136 Epub 20101122. 10.1186/1477-7525-8-136 21092189 10.1186/1477-7525-8-136 PMC3001456

[31] Jozefiak T, Larsson B, Wichstrøm L, Mattejat F, Ravens-Sieberer U (2008) Quality of life as reported by school children and their parents: a cross-sectional survey. Health Qual Life Outcomes 6:34 Epub 20080519. 10.1186/1477-7525-6-34 18489777 10.1186/1477-7525-6-34 PMC2409303

[32] Aanondsen CM, Jozefiak T, Heiling K, Lydersen S, Rimehaug T (2021) Psychometric properties of the inventory of life quality in children and adolescents in Norwegian sign language. BMC Psychol 9(1):89. 10.1186/s40359-021-00590-x. (**Epub 20210527**) 34044895 10.1186/s40359-021-00590-x PMC8161577

[33] Wolf K, Schmitz J (2023) Scoping review: longitudinal effects of the COVID-19 pandemic on child and adolescent mental health. Eur Child Adolesc Psychiatry. 10.1007/s00787-023-02206-8 37081139 10.1007/s00787-023-02206-8 PMC10119016

[34] Potrebny T, Nilsen SA, Bakken A, von Soest T, Kvaløy K, Samdal O et al (2024) Secular trends in mental health problems among young people in Norway: a review and meta-analysis. Eur Child Adolesc Psychiatry. Epub 20240216. 10.1007/s00787-024-02371-4. PubMed PMID: 38363391 10.1007/s00787-024-02371-4 PMC11805846 38363391

[35] Chen Y, Osika W, Henriksson G, Dahlstrand J, Friberg P (2022) Impact of COVID-19 pandemic on mental health and health behaviors in Swedish adolescents. Scand J Public Health 50(1):26–32 Epub 20210608. doi: 10.1177/14034948211021724. PubMed PMID: 34100665; PubMed Central PMCID: PMCPMC8808000 34100665 10.1177/14034948211021724 PMC8808000

[36] Myhr A, Naper LR, Samarawickrema I, Vesterbekkmo RK (2021) Impact of COVID-19 pandemic lockdown on mental well-being of Norwegian adolescents during the first wave-socioeconomic position and gender differences. Front Public Health 9:717747. 10.3389/fpubh.2021.717747. (**Epub 20210914**) 34595148 10.3389/fpubh.2021.717747 PMC8476849

[37] Parlikar N, Kvaløy K, Strand LB, Espnes GA, Moksnes UK (2023) Loneliness in the Norwegian adolescent population: prevalence trends and relations to mental and self-rated health. BMC Psychiatry 23(1):895 Epub 20231130. 10.1186/s12888-023-05404-5 38037032 10.1186/s12888-023-05404-5 PMC10688064

[38] Qualter P, Brown SL, Rotenberg KJ, Vanhalst J, Harris RA, Goossens L et al (2013) Trajectories of loneliness during childhood and adolescence: predictors and health outcomes. J Adolesc 36(6):1283–1293. 10.1016/j.adolescence.2013.01.005 23465384 10.1016/j.adolescence.2013.01.005

[39] Abio A, Owusu PN, Posti JP, Bärnighausen T, Shaikh MA, Shankar V et al (2022) Cross-national examination of adolescent suicidal behavior: a pooled and multi-level analysis of 193,484 students from 53 LMIC countries. Soc Psychiatry Psychiatr Epidemiol 57(8):1603–1613 Epub 20220421. 10.1007/s00127-022-02287-x 35445842 10.1007/s00127-022-02287-x PMC9288956

[40] Blázquez-Fernández C, Lanza-León P, Cantarero-Prieto D (2023) A systematic review on suicide because of social isolation/and loneliness: does COVID-19 make a difference? J Public Health (Oxf) 45(3):680–688. 10.1093/pubmed/fdad001 36680431 10.1093/pubmed/fdad001

[41] Hards E, Loades ME, Higson-Sweeney N, Shafran R, Serafimova T, Brigden A et al (2022) Loneliness and mental health in children and adolescents with pre‐existing mental health problems: a rapid systematic review. Br J Clin Psychol 61(2):313–334. 10.1111/bjc.12331 34529837 10.1111/bjc.12331

[42] Xerxa Y, Rescorla LA, Shanahan L, Tiemeier H, Copeland WE (2023) Childhood loneliness as a specific risk factor for adult psychiatric disorders. Psychol Med 53(1):227–235. 10.1017/s0033291721001422 34120674 10.1017/S0033291721001422 PMC9874978

[43] Amran MS, Jamaluddin KA (2022) Adolescent screen time associated with risk factor of fear of missing out during pandemic COVID-19. Cyberpsychol Behav Soc Netw 25(6):398–403 Epub 20220520. doi: 10.1089/cyber.2021.0308. PubMed PMID: 35594262 35594262 10.1089/cyber.2021.0308

[44] Bu F, Steptoe A, Fancourt D (2020) Loneliness during a strict lockdown: trajectories and predictors during the COVID-19 pandemic in 38,217 United Kingdom adults. Soc Sci Med 265:113521. 10.1016/j.socscimed.2020.113521 33257177 10.1016/j.socscimed.2020.113521 PMC7768183

[45] Bu F, Steptoe A, Fancourt D (2020) Who is lonely in lockdown? Cross-cohort analyses of predictors of loneliness before and during the COVID-19 pandemic. Public Health 186:31–34 Epub 20200805. 10.1016/j.puhe.2020.06.036 32768621 10.1016/j.puhe.2020.06.036 PMC7405905

[46] Hansen T, Nilsen TS, Yu B, Knapstad M, Skogen JC, Vedaa Ø et al (2021) Locked and lonely? A longitudinal assessment of loneliness before and during the COVID-19 pandemic in Norway. Scand J Public Health 49(7):766–773 Epub 20210301. doi: 10.1177/1403494821993711. PubMed PMID: 33645336 33645336 10.1177/1403494821993711

[47] Ahmed N, Barnett P, Greenburgh A, Pemovska T, Stefanidou T, Lyons N et al (2023) Mental health in Europe during the COVID-19 pandemic: a systematic review. Lancet Psychiatry 10(7):537–556 Epub 20230612. 10.1016/s2215-0366(23)00113-x 37321240 10.1016/S2215-0366(23)00113-X PMC10259832

[48] Aranda Z, Rodríguez-Cuevas FG (2021) COVID-19 and global mental health. Lancet Psychiatry 8(6):457. 10.1016/s2215-0366(21)00124-3 34023012 10.1016/S2215-0366(21)00124-3 PMC9764867

[49] Kauhanen L, Wan Mohd Yunus WMA, Lempinen L, Peltonen K, Gyllenberg D, Mishina K et al (2023) A systematic review of the mental health changes of children and young people before and during the COVID-19 pandemic. Eur Child Adolesc Psychiatry 32(6):995–1013. 10.1007/s00787-022-02060-0 10.1007/s00787-022-02060-0 PMC9373888 35962147

[50] Gustafsson J, Lyyra N, Jasinskaja-Lahti I, Simonsen N, Lahti H, Kulmala M et al (2023) Mental health profiles of Finnish adolescents before and after the peak of the COVID-19 pandemic. Child Adolesc Psychiatry Ment Health 17(1):54 Epub 20230429. 10.1186/s13034-023-00591-1 37120557 10.1186/s13034-023-00591-1 PMC10148589

[51] Meyer D, Sumner PJ, Tan EJ, Neill E, Hielscher E, Blake JA et al (2023) Comparing the impact of high versus low lockdown severity on the mental health of young people in Australia during the COVID-19 pandemic. Psychiatry Res 322:115121 PubMed PMID: 36854222; PubMed Central PMCID: PMCPMC9946783 36854222 10.1016/j.psychres.2023.115121 PMC9946783

[52] van den Boom W, Marra E, van der Vliet N, Elberse J, van Dijken S, van Dijk M et al (2023) General mental health, loneliness, and life satisfaction in the context of COVID-19 policies: a 2-year cohort study in the Netherlands, April 2020-January 2022. Public Health Rep 138(5):812–821 (**Epub 20230705. doi: 10.1177/00333549231176000. PubMed PMID: 37408335; PubMed Central PMCID: PMCPMC10323514**) 37408335 10.1177/00333549231176000 PMC10323514

[53] Fancourt D, Steptoe A, Bu F (2021) Trajectories of anxiety and depressive symptoms during enforced isolation due to COVID-19 in England: a longitudinal observational study. Lancet Psychiatry 8(2):141–149. 10.1016/s2215-0366(20)30482-x 33308420 10.1016/S2215-0366(20)30482-X PMC7820109

[54] Dregan A, Armstrong D (2023) Shifts in patterns of mental health burden during the COVID-19 pandemic. Lancet Reg Health Eur 32:100711 Epub 20230808. doi: 10.1016/j.lanepe.2023.100711. PubMed PMID: 37671128; PubMed Central PMCID: PMCPMC10477034 37671128 10.1016/j.lanepe.2023.100711 PMC10477034

[55] Yoo N, Jang SH (2023) Perceived household financial decline and physical/mental health among adolescents during the COVID-19 crisis: focusing on gender differences. Prev Med Rep 32:102119 Epub 20230124. 10.1016/j.pmedr.2023.102119 36718194 10.1016/j.pmedr.2023.102119 PMC9872569

[56] Argabright ST, Tran KT, Visoki E, DiDomenico GE, Moore TM, Barzilay R (2022) COVID-19-related financial strain and adolescent mental health. Lancet Reg Health Am 16:100391 Epub 20221115. 10.1016/j.lana.2022.100391 36405885 10.1016/j.lana.2022.100391 PMC9664255

[57] Blackwell CK, Mansolf M, Sherlock P, Ganiban J, Hofheimer JA, Barone CJ et al (2022) Youth well-being during the COVID-19 pandemic. Pediatrics. 10.1542/peds.2021-054754 35301542 10.1542/peds.2021-054754 PMC9169239

[58] Kim B, Kim DH, Jang SY, Shin J, Lee SG, Kim TH (2022) Family economic hardship and adolescent mental health during the COVID-19 pandemic. Front Public Health 10:904985 Epub 20220906. 10.3389/fpubh.2022.904985 36148341 10.3389/fpubh.2022.904985 PMC9486021

[59] Cyranowski JM, Frank E, Young E, Shear MK (2000) Adolescent onset of the gender difference in lifetime rates of major depression: a theoretical model. Arch Gen Psychiatry 57(1):21–27. 10.1001/archpsyc.57.1.21PubMed PMID: 10632229 10632229 10.1001/archpsyc.57.1.21

[60] Shih JH, Eberhart NK, Hammen CL, Brennan PA (2006) Differential exposure and reactivity to interpersonal stress predict sex differences in adolescent depression. J Clin Child Adolesc Psychol 35(1):103–115. 10.1207/s15374424jccp3501_9 16390306 10.1207/s15374424jccp3501_9

[61] Aasan BEV, Lillefjell M, Krokstad S, Sund ER (2024) Trends in social inequality and how mental wellbeing vary and covary among Norwegian adolescents and their families: the Young-HUNT study. Scand J Public Health 52:861–867. 10.1177/14034948231172634 37776173 10.1177/14034948231172634 PMC11476489

[62] Eriksen IM (2021) Class, parenting and academic stress in Norway: middle-class youth on parental pressure and mental health. Discourse Stud Cult Politics Educ 42(4):602–614. 10.1080/01596306.2020.1716690

[63] Högberg B, Strandh M, Hagquist C (2020) Gender and secular trends in adolescent mental health over 24 years - the role of school-related stress. Soc Sci Med 250:112890. 10.1016/j.socscimed.2020.112890 32143086 10.1016/j.socscimed.2020.112890

[64] Nygård M, Bjordal I (2024) School refusal and flight from the public school. Norsk Sosiologisk Tidsskrift 8(2–3):23–37. 10.18261/nost.8.2-3.3

[65] Havik T, Ingul JM (2021) How to understand school refusal. Front Educ. 10.3389/feduc.2021.715177

[66] Holmen TL, Bratberg G, Krokstad S, Langhammer A, Hveem K, Midthjell K et al (2014) Cohort profile of the Young-HUNT study, Norway: a population-based study of adolescents. Int J Epidemiol 43(2):536–544. 10.1093/ije/dys232 23382364 10.1093/ije/dys232

[67] Langhammer A, Krokstad S, Romundstad P, Heggland J, Holmen J (2012) The HUNT study: participation is associated with survival and depends on socioeconomic status, diseases and symptoms. BMC Med Res Methodol 12:143 Epub 20120914. 10.1186/1471-2288-12-143 22978749 10.1186/1471-2288-12-143 PMC3512497

[68] von Soest T, Wichstrøm L (2014) Secular trends in depressive symptoms among Norwegian adolescents from 1992 to 2010. J Abnorm Child Psychol 42(3):403–415. 10.1007/s10802-013-9785-1PubMed PMID: 23888312 23888312 10.1007/s10802-013-9785-1

[69] Quon EC, McGrath JJ (2014) Subjective socioeconomic status and adolescent health: a meta-analysis. Health Psychol 33(5):433–447. 10.1037/a0033716 24245837 10.1037/a0033716 PMC5756083

[70] Garbarski D (2010) Perceived social position and health: is there a reciprocal relationship? Soc Sci Med 70(5):692–699 Epub 20091216. doi: 10.1016/j.socscimed.2009.11.007. PubMed PMID: 20006415; PubMed Central PMCID: PMCPMC2841561 20006415 10.1016/j.socscimed.2009.11.007 PMC2841561

[71] Singh-Manoux A, Marmot MG, Adler NE (2005) Does subjective social status predict health and change in health status better than objective status? Psychosom Med 67(6):855–861. 10.1097/01.psy.0000188434.52941.a0 16314589 10.1097/01.psy.0000188434.52941.a0

[72] Glendinning A, Love JG, Hendry LB, Shucksmith J (1992) Adolescence and health inequalities: extensions to Macintyre and West. Soc Sci Med 35(5):679–87. 10.1016/0277-9536(92)90006-c 1439918 10.1016/0277-9536(92)90006-c

[73] Mund M, Maes M, Drewke PM, Gutzeit A, Jaki I, Qualter P (2023) Would the real loneliness please stand up?? The validity of loneliness scores and the reliability of single-item scores. Assessment 30(4):1226–1248. 10.1177/10731911221077227 35246009 10.1177/10731911221077227 PMC10149889

