# Supplementary material for: Adolescents’ health and well-being into the COVID-19 pandemic: A two-wave prospective investigation– The HUNT study
Source: Soc Psychiatry Psychiatr Epidemiol. 2025 Aug 18;61(4):723–34. doi: 10.1007/s00127-025-02978-1 (PMC13021855; doi:10.1007/s00127-025-02978-1)
Supplement: Supplementary file 1 — Supplementary Material 1 [file 127_2025_2978_MOESM1_ESM.pdf]

Article title: Adolescents' health and well-being into the COVID-19 pandemic: A two-wave prospective investigation – The HUNT Study

Journal name: Social Psychiatry and Psychiatric Epidemiology

Author names and affiliations: Kirsti Kvaløy[1,2,3]*, Erik Reidar Sund[1,3], Tormod Rimehaug [4], Kristine Pape[5], Jo Magne Ingul[4], Vegar Rangul[1,3].

[1] HUNT Research Centre, Department of Public Health and Nursing, Faculty of Medicine and Health Sciences, Norwegian University of Science and Technology, NTNU, Norway
[2] Centre for Sami Health Research, Department of Community Medicine, Faculty of Health Sciences, UiT The Arctic University of Norway, Tromsø, Norway
[3] Levanger Hospital, Nord-Trøndelag Hospital Trust, Levanger, Norway
[4] Regional Centre for Child and Youth Mental Health and Child Welfare, Department of Mental Health, Faculty of Medicine and Health Sciences, NTNU – Norwegian University of Science and Technology, Trondheim, Norway
[5] Department of Public Health and Nursing, Faculty of Medicine and Health Sciences, Norwegian University of Science and Technology, NTNU, Norway

Supplemental information

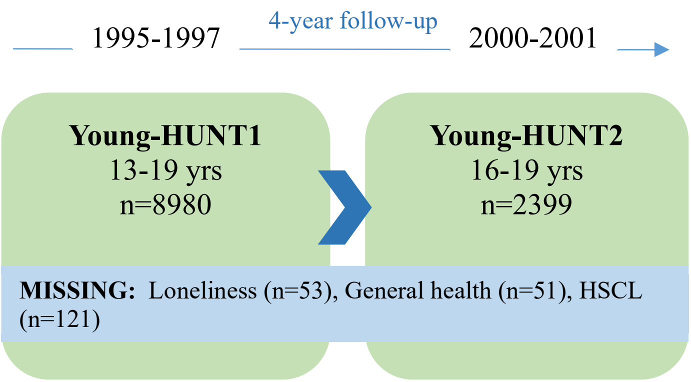

Fig S1 Flow chart of the study populations, Young-HUNT1 (1995-1997) and follow-up Young-HUNT2 (2000-2001), the longitudinal dataset consists of 2399 participants

Table S1. The association between low socioeconomic position (family affluence) and mental health, life quality (linear regression), loneliness and poor general health (logistic regression) in the combined Young-HUNT4 and Young-HUNT COVID samples.

	**Model 1**			**Model 2**		
**Outcome**	**B**	**95% CI**	**P value**	**B**	**95% CI**	**P value**
Mental health	4.09	3.55,4.64	< 0.001	4.1	3.50,4.60	< 0.001
Life quality	3.30	2.91,3.70	< 0.001	3.30	2.90,3.69	< 0.001
	**OR**	**95% CI**	**P value**	**OR**	**95% CI**	**P value**
Loneliness	2.85	2.33,3.49	< 0.001	2.88	2.37,3.50	< 0.001
Poor general health	3.01	2.51,3.62	< 0.001	2.93	2.45,3.51	< 0.001

Model 1: Adjusted for sex and age. Model 2: Adjusted for sex, age and survey participation (Young-HUNT4 or Young-HUNT COVID). Mental distress (HSCL-10 sumscore), life quality (ILC, sumscore), loneliness (Often/Very often), general health (Poor/Very poor).

Table S2. Prevalence in loneliness, poor general health, mental distress and poor life quality in the Young-HUNT4, YH4 (2017-2019) and Young-HUNT COVID, YHC (2021), stratified by sex and socioeconomic position (self-reported family affluence).

	YH4						YHC					
	High SEP			Low SEP			High SEP			Low SEP		
**Boys**	Case	Total no.	P (case/total)	Case	Total no.	P (case/total)	Case	Total no.	P (case/total)	Case	Total no.	P (case/total)
Lonely	137	1851	0.07	30	169	0.18	67	725	0.09	22	77	0.29
Poor general health	239	1916	0.12	44	175	0.25	87	741	0.12	21	80	0.26
Mental distress	268	1922	0.14	51	176	0.29	146	743	0.20	40	80	0.50
Poor life quality	479	1740	0.28	82	157	0.52	143	517	0.28	32	46	0.70
**Girls**												
Lonely	281	1896	0.15	76	233	0.33	185	1044	0.18	51	137	0.37
Poor general health	344	1939	0.18	105	244	0.43	183	1060	0.17	52	139	0.37
Mental distress	813	1945	0.42	162	244	0.66	477	1060	0.45	89	140	0.64
Poor life quality	439	1796	0.24	126	223	0.57	233	815	0.29	47	90	0.52

P = Prevalence. SEP = Socioeconomic position (self-reported family affluence). Lonely (Often/Very often), Poor mental health (Poor/Very poor), Mental distress (HSCL-10 ≥ 1.85), Poor life quality (ILC, sex-specific 25% upper percentile cut-offs; ≥ 8 in boys and ≥ 11 in girls).

Table S3. Comparisons of the Young-HUNT4 (2017-2019) and Young-HUNT COVID (2021) longitudinal dataset employing random effects panel models to examine changes in outcomes across survey waves, between sexes, including interaction terms for wave and sex – without (Model 1) and with (Model 2) adjustment for time-varying age.

	**Model 1**				**Model 2**			
	**Boys**		**Girls**		**Boys**		**Girls**	
**Variables**	**Coef.* (95% CI)**	**P**	**Coef.* (95% CI)**	**P**	**Coef.* (95% CI)**	**P**	**Coef.* (95% CI)**	**P**
Poor family affluence	0.82 (0.23, 1.41)	0.006	0.69 (0.30, 1.09)	0.001	- 0.64 (- 1.55, 0.27)	0.170	- 0.78 (- 1.59, 0.04)	0.063
Loneliness	0.64 (0.11, 1.17)	0.017	0.83 (0.53, 1.12)	< 0.001	- 0.43 (- 1.17, 0.30)	0.249	- 0.26 (- 0.83, 0.32)	0.380
Poor general health	0.11 (- 0.30, 0.53)	0.586	0.84 (0.53, 1.15)	< 0.001	- 0.84 (- 1.51, - 0.17)	0.014	- 0.12 (- 0.72, 0.49)	0.705
HSCL: sumcore	1.46 (0.96, 1.96)	< 0.001	2.85 (2.35, 3.35)	< 0.001	- 1.62 (- 2.64, - 0.60)	0.002	- 0.24 (- 1.32, 0.84)	0.663
ILC: sumscore	0.45 (0.07, 0.84)	0.019	1.99 (1.69, 2.30)	< 0.001	- 1.15 (- 1.89, - 0.41)	0.002	0.38 (- 0.32, 1.08)	0.287

* Coefficient for poor family affluence, loneliness and poor general health is the log-odds between waves (YH4 and YHC).

Table S4. Characterisation and sex-stratified comparison of the two age groups, 13–15-year-olds and 16–19-year-olds, in Young-HUNT4 (2017-2019).

	Boys (n=3960)			Girls (n=4106)		
**Variables**	13-15y (n=1825)	16-19y (n=2135)	P value *	13-15y (n=1894)	16-19y (n=2212)	P value *
Age: Mean, SD	14.40 (0.9)	17.54 (1.0)		14.44 (0.8)	17.52 (0.9)	
Family economy: Low, n (%)	78 (4.4)	176 (8.4)	<0.001	131 (7.0)	244 (11.1)	<0.001
Loneliness: Often/Very often, n (%)	79 (4.6)	171 (8.4)	<0.001	207 (11.3)	362 (16.9)	<0.001
General health: Poor/Very poor, n (%)	160 (8.9)	286 (13.5)	<0.001	217 (11.6)	455 (20.7)	<0.001
HSCL sumscore, SD	13.27 (4.2)	14.24 (5.0)	<0.001	16.96 (6.6)	19.46 (7.4)	<0.001
ILC sumscore, SD	5.27 (4.2)	5.65 (4.2)	0.008	6.47 (4.5)	7.99 (4.6)	<0.001

*T-test - two-sided (continuous). Pearson Chi-Square (asymptotic significance 2-sided)

Table S5. Characterisation and sex-stratified comparison of the Young-HUNT1, YH1 (1995-1997) (13–15-year-olds) and Young-HUNT2, YH2 (2000-2001) (16–19-year-olds) longitudinal dataset.

	Boys (n=1115)			Girls (n=1284)		
**Variables**	**YH1**	**YH2**	**P value***	**YH1**	**YH2**	**P value ***
Age: Mean, SD	14.5 (0.9)			14.6 (0.9)	18.4 (0.8)	
Loneliness: Often/Very often, n (%)	37 (3.4)	55 (5.0)	0.066	67 (5.3)	104 (8.2)	0.004
General health: Poor/Very poor, n (%)	80 (7.3)	113 (10.2)	0.005	105 (8.3)	178 (14)	<0.001
HSCL-5, sumscore (SD)	6.38 (1.9)	6.90 (2.3)	<0.001	7.22 (2.3)	8.35 (2.9)	<0.001

*Friedman test.

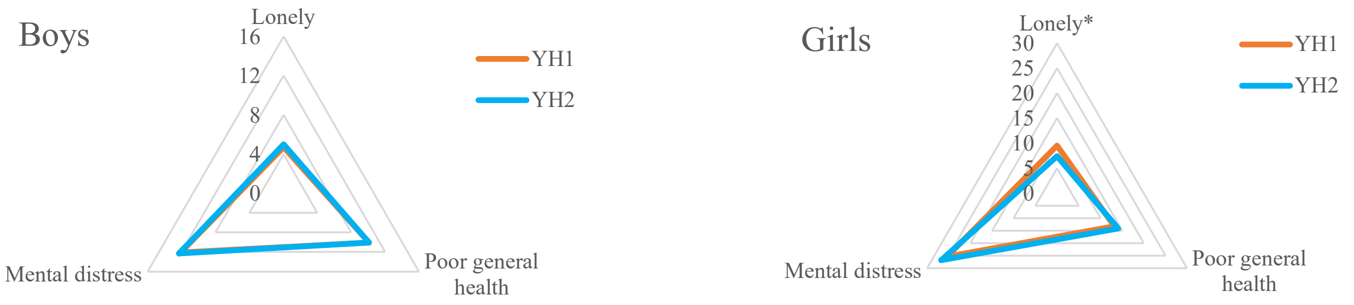

Fig S2 Proportions (%) of participants reporting loneliness, poor general health, and mental distress in the two cross-sections: Young-HUNT1, YH1 (1995-1997) and Young-HUNT2, YH2 (2000-2001) in boys and girls. * P value < 0.05 indicating statistically significant difference between YH1 and YH2
